# LIMP Score and Anterior Cruciate Ligament Injury in Patient Undergoing Arthoscopy for Acute Knee Injury: An Observational Study

**DOI:** 10.31729/jnma.8858

**Published:** 2025-01-31

**Authors:** Mahesh Karmacharya, Binod Sherchan, Bikash KC, Ram Sharma Subedi, Badri Rijal, Saphalata Devi Koju

**Affiliations:** 1 Department of Orthopedics, National Academy of Medical Sciences, Mahaboudha, Kathmandu, Nepal; 2 Bhaktapur Hospital, Dudhpati, Bhaktapur, Nepal

**Keywords:** *ACL injury*, *arthroscopy*, *history based diagnosis*, *LIMP score*

## Abstract

**Introduction:**

Globally, anterior cruciate ligament injury is common but difficult clinical diagnosis in acute setting because of pain during examination and difficult availability of investigations and diagnostic arthroscopy. Legs giving way, Inability to continue the task, Massive effusion and pop sound heard at the time of injury or LIMP score is a history based scoring which can be aid to diagnose anterior cruciate ligament injury. This study is designed to find out the proportion of patient with LIMP score of >2 having anterior cruciate ligament injury.

**Methods:**

An observational cross-section study was done among the patients visiting emergencies and out patient department of respective hospitals after approval from Institutional review Committee (Reference number: 23/2078/79). The LIMP score was assessed during the presentation and patients were followed up at arthoscopy for the final diagnosis.

**Results:**

There were 126 patients enrolled in the study among which 68 (54%) had LIMP score of >2. Out of the total, 74 (59%) had anterior cruciate ligament inury among which 67 (90.5%) had LIMP score >2. Among the patient who did not have anterior cruciate ligament injury, 51 (98%) had LIMP score <2.

**Conclusions:**

The proportion of patients having anterior cruciate ligament injury were higher in the category with LIMP score >2.

## INTRODUCTION

Anterior cruciate ligament (ACL) injury accounts for a global burden of estimated 1 million annually,^1^ and is one of the common ligament injuries.^2,3^ Clinical diagnosis has limitation in acute setting because of the pain with diagnostic accuracy ranging 6.8% to 28.2%.^4^ Many typical history findings were documented in the literature,^5-8^ and LIMP score is history based questionnaire comprising of Leg giving way, Inability to continue the task, Massive swelling, and Pop sound heard or felt at the time of injury with results of LIMP score >2 and ACL injury in as much as 95.2%.^1^

The LIMP score is based on history rather than examination so can be comfortably used to aid diagnosis of ACL injury without painful clinical examination.^9,10^

The aim of this study is to find the proportion of patient with ACL injury in LIMP score >2.

## METHODS

This was an observational cross-section study carried out at National Trauma Center, Civil Service Hospital and Bharatpur Hospital. These were tertiary care hospitals located at Kathmandu and Chitwan, Nepal. The study was conducted from April 2021 to March 2024 after ethical approval from Institutional Review Committee (Reference number: 23/2078/79). Data were collected after informed written consent from the participants.

All patient with history of acute knee injury within three weeks duration presenting to Emergency and Out Patient Department (OPD) of the respective hospitals, who were planned for knee arthoscopy were included in the study. Participants with fracture or dislocations, open injuries, multi-ligament injuries, those who didn't undergo arthroscopy, and patients refusing the study were excluded.

The patients were enrolled at Emergency or OPD and followed up till arthoscopic procedure. Demographic details and LIMP score were collected at presentation and the final diagnosis was recorded after arthoscopy. Patients were categorized into group based on LIMP score <2 and >2.

The data collected was entered in Microsoft Excel 2016 and descriptive analysis was done using SPSS Statistics for Windows, version 16.0 (SPSS Inc, Chicago, Ill., USA).

## RESULTS

A total of 126 patients with acute knee injuries were identified in the emergency department and OPD who were later followed up for arthroscopy.

The demographic analysis of the result showed male predominance of 81 (64.29%) and the most common age group to participate in the study to be the age group of 21-30 with 50 (39.68%). Majority of the patients had right sided knee injuries comprising 87 (69.05%) of the total (Table 1).

Stratification of LIMP score was done in a tabulated form which showed majority of the patient had LIMP score of 1 comprising 58 (46.03%) and the least being LIMP score 4 comprising 4 (3.17%), (Table 2).

**Table 1:** Demographic profile of patient with acute knee injury (n=126).

n (%)		
Sex	Male	81 (64.29)
	Female	45 (35.71)
Age Group	11-20	21 (16.66)
	21-30	50 (39.68)
	31-40	35 (27.78)
	41-50	13 (10.32)
	51-60	6 (4.76)
	>60	1 (0.79)
Side	Right	87 (69.05)
	Left	39 (30.95)

**Table 2:** Distribution based on LIMP score in patient with acute knee injury (n=126).

LIMP Score	n (%)
LIMP Score 1	58 (46.03)
LIMP Score 2	48 (38.09)
LIMP Score 3	16 (12.69)
LIMP Score 4	4 (3.17)

LIMP=Leg giving way, Inability to continue the task, Massive swelling and Pop sound

Further stratification of the LIMP score into groups with LIMP score> 2 and LIMP score <2 showed 58 (46.03%) belonged to the group LIMP score >2 (Table 3).

**Table 3:** Stratification of acute knee injury (n=126).

**LIMP score** <2 >2	**n (%)** 58 (46.03) 68 (53.97)

LIMP=Leg giving way, Inability to continue the task, Massive swelling and Pop sound

Among the total patient undergoing arthroscopy 74 (58.73%) had ACL injury and 52 (41.27%) did not have ACL injury (Table 4).

**Table 4:** Arthroscopic findings in patient with acute knee injury (n=126)

ACL status	n(%)
ACL tear	74 (58.73)
No ACL tear	52 (41.27)
**Finding in Cases with No ACL Tear (n=52)**	
Meniscal injuries	51 (98.08)
Normal finding	1 (1.92)

ACL=Anterior Cruciate Ligament

A comparison of arthroscopic ACL injury with LIMP score >2 was done which found 67 (98.52%) out of 68 with LIMP score >2 had arthroscopic ACL injury and 51 (87.93%) out of 58 with LIMP score >2 had no ACL injury in arthroscopy (Table 5)

**Table 5:** Comparison of LIMP score with arthroscopic ACL injury (n=126)

**LIMP Score** LIMP >2 LIMP <2	**ACL injured** 67 (53.17%) 7 (5.55%)	**ACL not injured** 1(0.80%) 51(40.48%)

LIMP=Leg giving way, Inability to continue the task, Massive swelling and Pop sound; ACL=Anterior Cruciate Ligament

## DISCUSSION

This study is a prospective observational study done in multicentric tertiary hospitals of Nepal hoping to include the patients from different parts of the country. Diagnosis has always been a challenge in our context because of poor advanced diagnostic modalities in our setup especially in the majority of the remote regions. Further acute knee injuries is associated with pain which limits the proper examination of the knee in the early injuries.^4,11^ Early diagnosis has always been concerns as late diagnosis of ACL injury has been associated with altered knee kinematics after injury^7^ and substantial secondary injuries to the meniscus which leads to early joint degenerations.^4,12-14^ The result of which has lead ACL injury as a public health burden.^6,15^ Many typical history findings were documented in the literature^5^ taking different clinical parameters including clinical examination but LIMP score is history based questionnaire comprising of leg giving way, inability to continue the task, massive swelling and pop sound heard or felt at the time of injury with results of LIMP score >2 and ACL injury in as much as 95.2 %.^1^

LIMP score is based on history rather than examination so can be comfortably used to aid diagnosis of ACL injury without painful clinical examination.^9,10^ Further in a developing countries like Nepal where there is lack of health infrastructure in remote areas like magnetic resonance imaging and diagnostic arthroscopy, the LIMP score can be a valuable aid for early diagnosis of ACL injury. This study is expected to increase the diagnostic abilities by history taking and emphasizes on use of LIMP score as an aid to diagnosis of ACL injury using only history taking parameters.

Regarding demographic evaluation our study showed 81 (64.29%) of male dominance in the study with only 45 (35.71%) female participating in the study compared to 80.1% predominance of male in the study done by Colin Ayre et al^1^. Similarly the most common age group is found to be in the age group of 21-30 years with 50 (39.68%), followed by 31-40 years 35 (27.78%), and 1120 years 21 (16.66%), 41-50 years 13 (10.32%), 51-60 years 6 (4.76%) and >60 years 1 (0.80%). Comparing to Colin Ayre et al the commonest age group being 20-29 years1. But in the study done by Gianotti et al the maximum involved age group involved both 2029 years and 50-59 years age^2^ group which was in contrast to 6 (4.76%) involvement of the age group of 50-59 years in our study. The reason is attributable to improper diagnosis in the age group of 50-59 years with osteoarthritis and degenerative changes in the age group of 50-59 years and poor reporting of the age group 50-59 to the hospital for the management of ligamentous injury.

Among the participants most of the participants have right sided injuries 87 (69.05%) compared to 52 % right sided injuries in Wagemakers et al and 58.1% right sidedness in Kochhal et al^9,16,^ which is comparable to our study.

In our study the majority of the participants 58 (46.03%) have LIMP score of 1, followed by LIMP score of 2 in 48 (38.10%)) and LIMP score of 3 in 13% (n=6) and LIMP score of 4 in 4 (3.17%). Comparing to the study done by Colin Ayre et al (2017) which is a multicentric retrospective study done in United Kingdom, LIMP score of 4 was seen in 57.8 %^1^, which is in contrast to our study. The reason attributable to this is probably due to retrospective nature of the study. In a study done by Noyes et al in 1980 in the retrospective study 65% of the participants had typical 4 histories and on prospective study it was found to be 38% only to typical present with all 4 features. In the study done by Wagemakers et al 43% of the participants had typical history but lacks the inability to continue. Similary in a study done by Bollen et al typical history was seen in 52.3% despite the fact that inability to continue the activity was not included in the study.^12^ In the study done by Guillodo et al^17^ in 2018, 48.1% of the participants had typical history of leg giving way, swelling of knee and pop sound heard or felt at the time of injury but this study also lacks the history of inability to continue after injury.

The disparity of the results in our study may be attributable to the level of understanding, care during injury, mode of injury. While most of the injuries in above mentioned cases were sports related, our study includes road traffic accidents and fall injury which might have obscured our history. Further our study was a prospective study so patient without ACL injury may have lower LIMP score in contrast to retrospective studies where LIMP score of the patient with ACL injuries are only taken into account.

On stratification the group into <2 LIMP score and >2 LIMP score, the study shows the LIMP score of <2 in 58 (46.03%) and LIMP score >2 in 68 (53.97%) in comparison to 7% with LIMP score <2 group and 93 % with LIMP score >1 group in Colin Ayre et al^1^ which were contrasting. The disparity of the result may be contributed to collection of data which was prospective in our study and retrospective in their study and we have included both ACL injury and no ACL injury whereas Colin Ayre et al have included only patient with ACL injury.

Similar studies have been done in 2022 in France by S. Lukas et al^8^, with a sample size of 228, 4 parameters were taken based on history and 3 based clinical examination and MRI taken as diagnostic modalities.^8^

Compared to the study our study is based on gold standard arthroscopic diagnosis but with the sample size of 126 and only 4 history based parameters were taken into account. The parameters of clinical examination is omitted in our study because our study is based on LIMP score which is based on history taking alone.

There are always fallacies to some extent and history variability has been a limitation to this study especially in case of effusion. A necessary criterion should be validated to grade a massive effusion which is lacking in our study. Despite being a multicentric study around the country, all of the centers are tertiary center of the country so inclusion of the patient from the remote who cannot get referred to the tertiary center as missed. So despite being inclusive sample the study lacks the sample from the remote areas where we were supposed to be fruitful. Further the study sample is small to give a statistical correlation between LIMP score and arthroscopic ACL tear but the findings of this study can be used as guide in the future to study about prevalence, different LIMP score and its association with ACL tear.

## CONCLUSIONS

The proportion of patients having anterior cruciate ligament injury were higher in the category with LIMP score >2. In the patient with no ACL tear, meniscal injury was common.
